# Harvest Programs in First Nations of Subarctic Canada: The Benefits Go Beyond Addressing Food Security and Environmental Sustainability Issues

**DOI:** 10.3390/ijerph17218113

**Published:** 2020-11-03

**Authors:** Leonard J. S. Tsuji, Stephen R. J. Tsuji, Aleksandra M. Zuk, Roger Davey, Eric N. Liberda

**Affiliations:** 1 Department of Physical and Environmental Sciences, University of Toronto, Toronto, ON M1C 1A4, Canada; aleksandra.zuk@utoronto.ca; 2 School of Environmental Studies, Queen’s University, Kingston, ON K7L 3N6, Canada; srjt@queensu.ca; 3 School of Nursing, Faculty of Health Sciences, Queen’s University, Kingston, ON K7L 3N6, Canada; 4 Fort Albany First Nation, Fort Albany, ON P0L 1H0, Canada; roger.daveyFAFN@gmail.com; 5 School of Occupational and Public Health, Ryerson University, Toronto, ON M5B 2K3, Canada; eric.liberda@ryerson.ca

**Keywords:** First Nations of subarctic Canada, food security, harvesting of overabundant species, sharing networks, Indigenous peoples’ perspective of being on the land, Indigenous knowledge transmission, strengthening of social networks, wellness

## Abstract

By breaking down barriers that impacted the ability of subarctic First Nations people to harvest waterfowl, the Sharing-the-Harvest program provided a safe, nutritious, and culturally appropriate food (i.e., geese) to James Bay Cree communities while also helping to protect the environment by harvesting overabundant geese. However, the impacts extend beyond those described above. Thus, the objectives of the present paper are twofold: to document the food sharing networks of the Sharing-the-Harvest program; and to examine the benefits associated with the harvest program beyond food security and environmental sustainability issues, as revealed through semi-directed interviews. In the regional initiative, harvested geese were shared with all James Bay communities; sharing is an important part of Cree culture. Where detailed information was collected, the goose-sharing network reached 76% of the homes in one of the communities. Likewise, in the local initiative, the goose-sharing network had a 76% coverage rate of the homes in the community. Although decreasing food insecurity was an important focus of the harvest-sharing programs, there were other benefits, from an Indigenous perspective, of being on the land, as identified by the Cree harvesters through semi-directed interviews (e.g., the transmission of Indigenous knowledge, the strengthening of social networks, and the feeling of wellness while out on-the-land). Thus, by participating in the on-the-land harvest programs, the Cree gained benefits beyond those solely related to strengthening food security and contributing in part to environmental sustainability. The Sharing-the-Harvest protocol has the potential to be adapted and employed by other Indigenous (or marginalized) groups worldwide, to help improve health and wellness, while, also protecting the environment from overabundant and/or invasive species.

## 1. Introduction

### 1.1. Food Security

Food security issues affect people worldwide, especially Indigenous populations [1,2,3,4,5,6]. When there is access to enough safe and nutritious food to meet the dietary needs and preferences of individuals to allow for an active and healthy lifestyle, food security is said to exist [7]. Food insecurity presents if these conditions are not met [8]. It is well documented that Indigenous Canadians, especially in remote communities, suffer disproportionately from food insecurity [6,9]. This is particularly true for the Cree people inhabiting the western James Bay region of northern Ontario, Canada [10,11,12], where the prevalence of household food insecurity has been reported to be as high as 70% [13]. The James Bay Cree diet is a mixture of store-bought and traditional foods (i.e., fish, game meat, etc.) [14]. Major barriers to the consumption of healthy foods in the western James Bay region include the following: the availability, quality, and high cost of store-bought foods; the expense associated with hunting and fishing; and the procurement of nutrient-dense game meats by the Cree [14,15,16]. Further, there are other factors affecting the consumption of traditional foods by James Bay Cree, such as climate change impacting fish health [17] and fish harvesting [18], as well as worry about environmental contamination of traditional meats [19]. Nonetheless, the Cree schoolchildren of James Bay would consume more traditional food if it was available at home [19].

It has been suggested that the most culturally appropriate interventions would come from the people themselves; thus, our research team asked the Cree for suggestions on how to improve community food security [16]. These suggestions informed our Sharing-the-Harvest program. Example interventions proposed by the Cree people included:

[There should be] scheduled hunting trips where gas and supplies are paid [by the Band, the locally-elected First Nation government] and traditional food/meat caught given to lower income families…Lots of great hunters and trappers in this community—utilize them.

[16]

Get Band Council to get some hunters to go hunting for spring and fall. Supply the hunters with guns, shells, gas for their trip. Whatever game [meat] is killed, it should be shared within the community.

[16]

As can be noted from the passages above, the sharing of traditional food is an important aspect of both suggestions, which is understandable because sharing of food is a socially and culturally-embedded activity in Canadian Indigenous communities [6,15,20]. It then follows that any type of potential food insecurity intervention should ideally incorporate the activity of sharing within its framework [16]. 

Taking into account that wild game and fish have important nutritional value [21,22], there still exists great concern among the people of James Bay [19,23,24,25] and throughout Canada [26,27] about the contamination of traditional foods. Thus, any traditional food harvesting and sharing program must ensure that the food harvested and shared meets food consumption guidelines for contaminants. 

### 1.2. Lead Contamination

Lead is a non-essential and toxic metal; any exposure to lead can be detrimental to human health [28]. In the United States of America (USA) and Canada, lead shotshell for waterfowl hunting has been banned [29,30]. However, the use of lead shotshell in Canada for the harvesting of upland game (e.g., birds, small mammals) [31,32] is still legal, and sometimes used for waterfowl hunting in remote areas [33,34]. The food security issue associated with the use of lead shotshell to harvest wild game relates to lead ammunition fragmenting and becoming embedded in the wild meat [35,36,37,38]. In several studies, ~10% of game birds harvested with lead ammunition were above the Canadian consumption guideline for lead in protein (0.5 μg/g wet weight) [37,39]. Further, there is radiographic evidence that the presence of lead pellets and/or fragments in the digestive system of James Bay Cree is a common occurrence [40]. These lead pellets and/or fragments located in the digestive tract of humans are not inert; the lead can become a chronic source of exposure [41] or even cause acute lead poisoning [42]. Noteworthy, elevated tissue-lead levels have been reported for Indigenous people worldwide, who consume wild game birds harvested with lead ammunition [43,44], including the James Bay Cree [40,45]. Moreover, lead ammunition has been definitively identified as a major source of lead for James Bay Cree using stable-lead-isotope ratios [46,47]. Thus, any harvest-sharing program with the James Bay Cree should only use non-lead ammunition (e.g., steel shotshell), so as to not lead contaminate the meat [32].

### 1.3. Lesser Snow Goose (Chen Caerulescens Caerulescens)

**Figure 1:**
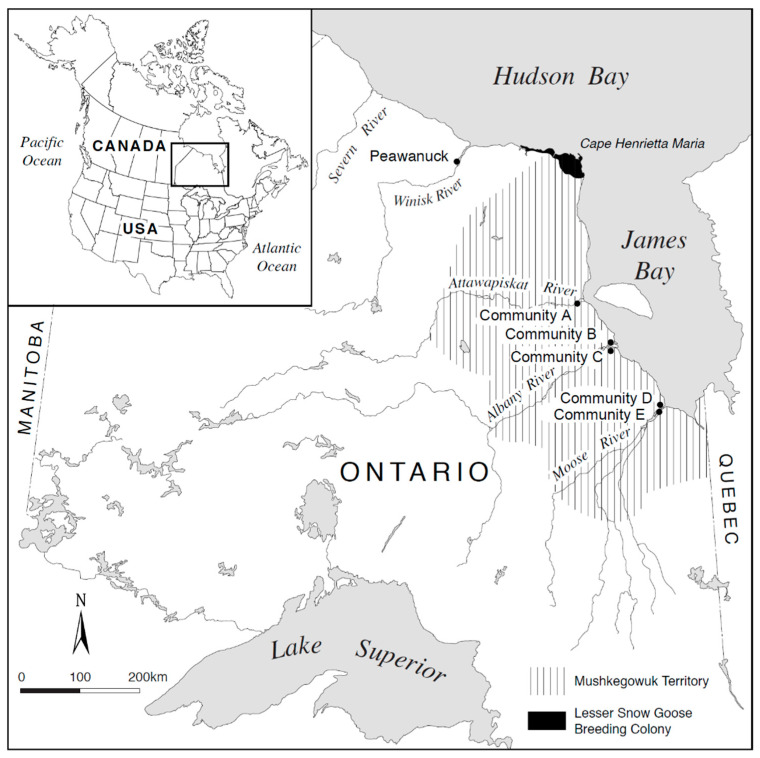
Map of the study region showing Cape Henrietta Maria and the western James Bay communities.

For over 50 years, the population of lesser snow geese breeding in the James and Hudson Bay region (Figure 1) has increased exponentially [48,49,50], due to anthropogenic reasons (e.g., global warming, refuge provisions) [49,50,51]. As early as the 1990s, researchers and wildlife managers realized that the time had come to manage the overpopulation of lesser snow geese that was destroying the environment through foraging behaviour; this behaviour destabilized the thin soil layer of the Hudson and James Bay region causing desertification [52,53]. A drastic departure from regular hunting regulations was required to protect goose habitat from future desertification and allow time for the recovery of the environment, and to save the snow geese (and other organisms) that inhabit the arctic and subarctic region [49,51,53]. In 1999, a joint effort by the Canadian and American governments resulted in a special spring hunt, as well as an increased number of geese that could be harvested by non-Indigenous hunters in the USA and Canada [49,51]. However, these changes have only slowed snow goose population growth [49,51], except at Cape Henrietta Maria, Ontario, Canada, where a decrease in lesser snow goose nests has been seen [51].

### 1.4. The Sharing-the-Harvest Intervention

#### 1.4.1. Implementation of the Program

**Figure 2:**
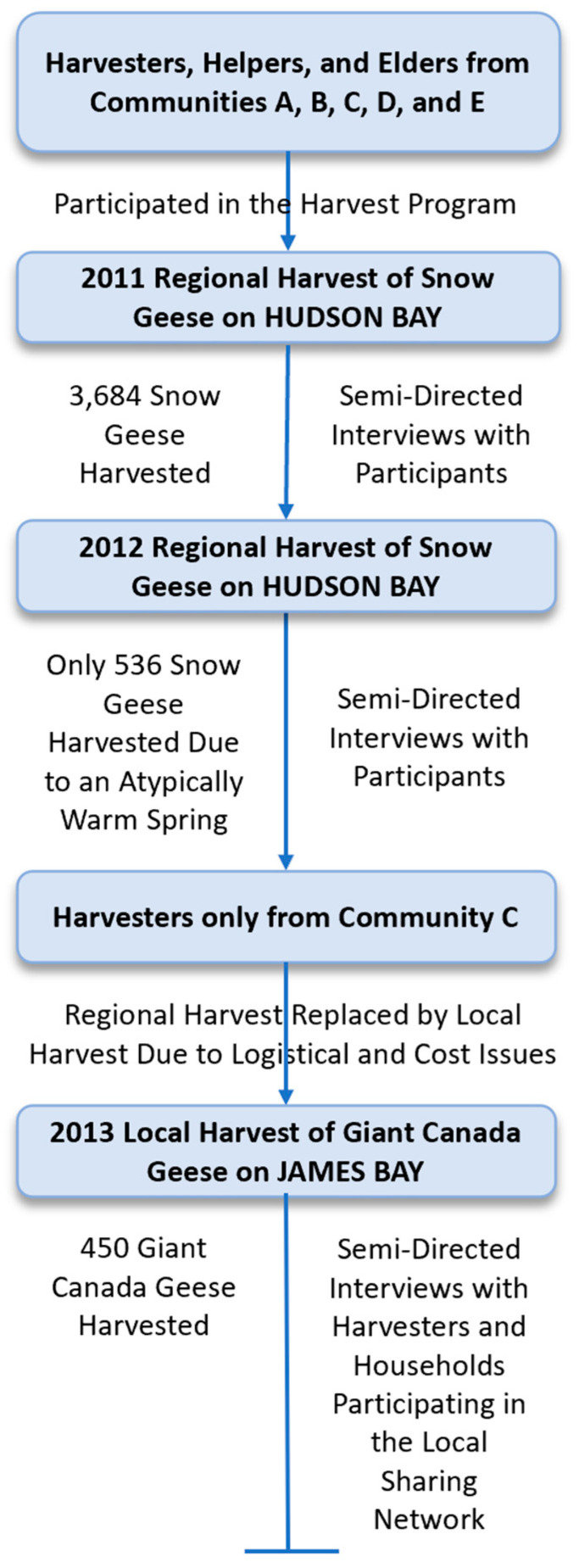
The evolution of the Sharing-the-Harvest program including the timing of data collection.

The regional Sharing-the-Harvest intervention provided assistance to harvesters, Elders, and helpers from the five western James Bay communities for air transportation to Cape Henrietta Maria, Hudson Bay, Ontario, Canada (Figure 1), and field transportation [32]. In addition, harvesters were supplied with materials to construct temporary camps, field supplies and other necessities [32]. Importantly, to minimize lead exposure during harvesting activities including the consumption of harvested meat—as well as to eliminate lead ammunition deposition into the environment—steel shotshell and the appropriate firearm for the safe use of this ammunition were supplied [32]. This assistance helped to address identified barriers to harvesting activity and facilitated the spring harvest of lesser snow geese. Further, with respect to human consumption, organochlorines and toxic metals [32,54] in snow geese from the western James Bay region [23,25] were not of concern. Nevertheless, after the spring 2012 harvest, the regional Sharing-the-Harvest initiative needed to evolve (Figure 2), because of the high costs of air transportation and the unpredictability of snow goose migration routes due to climate change [32,55]. The high cost of air transportation in the north [22] with respect to other lesser snow goose harvesting programs has been noted elsewhere [22]. 

With a suggestion from the Cree harvesters, the regional focus of the harvest program became local in scope [32] (Figure 2). A local focus allowed for the use of Indigenous knowledge to track in real-time goose movement in order to make better use of resources [32]. When harvesting locally, many eyes are watching for the geese, and harvesting locations can be changed rapidly based on Indigenous knowledge [32]. Harvesters also suggested that the molt-migrant giant Canada goose (*Branta canadensis maxima*) be harvested during early summer; these geese were also overabundant due to anthropogenic reasons [52] and negatively impacted the environment [56,57]. It should be emphasized that Canada geese harvested from the western James Bay region have been shown to be a relatively uncontaminated source of traditional meat [32], with respect to organochlorines [23,25] and toxic metals [54,58].

#### 1.4.2. Current State of Knowledge with Respect to the Harvest Program

Nutritionally speaking, the Sharing-the-Harvest intervention significantly contributed to increased intake of protein, vitamin B12, iron, and zinc in James Bay Cree youth [14]. Further, the use of steel shotshells meant that more spent leaded ammunition was not deposited in the environment [32,59]. Additionally, the intervention has contributed in part to the effort to try to protect the northern Canadian environment from further desertification, by harvesting overpopulated lesser snow geese and overpopulated giant Canada geese [32,55]. 

Finally, although data for goose harvest numbers and weights from the Sharing-the-Harvest intervention have been published previously [32,55], the sharing aspect of the program has never been examined. Likewise, semi-directed interview data with respect to improving the Sharing-the-Harvest intervention has been reported elsewhere [32], but impacts other than food security effects have not been explored.

### 1.5. Present Study Objectives

The present study objectives are twofold: (1) to examine the food sharing networks of the Sharing-the-Harvest program; and (2) to explore other benefits associated with the harvest program as revealed qualitatively by the semi-directed interviews. 

## 2. Materials and Methods 

### 2.1. The Study Area

The western James Bay and southwestern Hudson Bay region of northern Ontario, Canada—also known as the Mushkegowuk Territory—has a population of ~10,000 First Nations’ Cree (Figure 1). In the western James Bay region, the Cree inhabit five communities: one town and four remote First Nations [20,32]. Historically, the concept of the seasons was one of the cornerstones of the sustainable harvesting practices of James Bay Cree [20,32]. Each of the six seasons represented a period of time when one species (or a group of species) were plentiful and/or accessible; the Cree would only take enough for subsistence and sharing [20,32]. At present, the harvesting of waterfowl is still a way of life for the Cree, especially the spring harvest of the Canada goose, *B. canadensis interior* [20,32]. Furthermore, the Cree traditional diet of wild meats and fish has been shown to have significantly higher concentrations of protein and important amino acids, such as tryptophan—but lower concentrations of fat—compared to the modern processed diet [60]. Moreover, within the traditional diet food group, goose was reported to be significantly higher in protein when compared to moose (*Alces alces*) and fish [60].

### 2.2. Ethics

All activities were in keeping with the ethical standards of the University of Waterloo (ORE # 16534), Waterloo, Ontario, Canada. All applicable Government of Ontario and Government of Canada regulations with respect to waterfowl harvesting by First Nations peoples were followed. Informed consent was given by all participants in the study.

### 2.3. Data Collection

#### 2.3.1. Sharing-the-Harvest: A Regional Initiative

In the spring of 2011 and 2012, the species and number of snow geese harvested were recorded at Cape Henrietta Maria by Cree project coordinators (Figure 2). Although participants initially agreed that 50% of the harvested snow geese would be shared with the harvesters’ home communities, the sharing percentage was changed in the field by Cree project coordinators to a numerical value, to better accommodate and share the harvest along community affiliations. In 2011, detailed notes were taken about the sharing of geese at the community level, and in several communities we were able to able to collect end-point distribution data.

In 2011 and 2012, semi-directed interviews in Cree and/or English were conducted with participating harvesters either individually or in a group (Figure 2). Three general questions were asked of participants: how the program could be improved; whether there were any effects associated with the program; and how the program could be sustained. Interviews were digitally recorded and/or notes were taken, or written comments were submitted by the participants. Digital recordings were transcribed verbatim if in English and translated to English if in Cree.

#### 2.3.2. Sharing-the-Harvest: A Local Initiative

The local initiative, due to logistical and monetary factors, was focused in one western James Bay First Nation, Community C (Figure 2). In the summer of 2013, the species and number of giant Canada geese harvested were recorded. Detailed notes were taken of the distribution of the giant Canada geese shared in Community C.

In 2013, semi-directed interviews in Cree and/or English were conducted with participating harvesters, and the three general questions asked in the regional initiative were also used in the local initiative (Figure 2). Interviews were digitally recorded, and transcribed verbatim if in English, and translated to English if in Cree.

In Community C, the homeowners that received giant Canada geese were asked what their preference was between fresh versus smoked giant Canada geese. In addition, and of importance for the present study, homeowners were queried whether there were any effects associated with the Sharing-the-Harvest program.

### 2.4. Data Analyses

Separate flow charts for the two sharing initiatives recorded the number of geese shared in each community, down to the lowest level (i.e., the home) when possible. Qualitative data from the semi-directed interviews were organized into the regional and local sharing initiatives, and Community C homeowners. Qualitative data were then analyzed using a combination approach of deductive and inductive thematic coding [61,62]. The data were first deductively analyzed using a template organizing approach [61] where the two relevant interview questions for the present study—that is, whether there were any effects associated with the program, and how the program could be sustained—were used as a coding template. Subsequently, inductive thematic coding was employed to reveal additional insights [62].

## 3. Results

### 3.1. Participant Characteristics

#### 3.1.1. Sharing-the-Harvest: A Regional Initiative in the Cape Henrietta Maria Area, Western Hudson Bay Region, Ontario

In 2011 and 2012, a total of 73 unique individuals participated in the regional Sharing-the-Harvest initiative: 62 harvesters, and 11 helpers and Elders [32,55]. Of the 73 unique individuals, two were female and 71 were male. Participants were ≥18 years of age. Of the 73 unique participants, 66 were interviewed for a 90% (66/73) interview coverage rate; seven individuals were unavailable for interviews for a variety of reasons (e.g., out of town, not at home on multiple occasions). The results of the semi-directed interviews were discussed with the interviewees, either individually or in a group. This information was also shared with First Nation leadership (e.g., Chiefs, Councilors, and Health Directors).

#### 3.1.2. Sharing-the-Harvest: A Local Initiative in the Western James Bay Region, Ontario

In 2013, a total of 20 unique harvesters participated in the local Sharing-the-Harvest initiative [32]; number of helpers was not recorded and helpers were not interviewed. Of the 20 unique harvesters, all were males. Participants were ≥18 years of age. All 20 unique participants were interviewed for a 100% interview coverage rate. Results were discussed with the interviewees, either individually or in a group, and results were also shared with First Nation leadership (e.g., Chief, Councilors, and Health Director).

#### 3.1.3. Households Participating in the Local Sharing-the-Harvest Program

In 2013 in Community C, a total of 109 homes received geese from the local Sharing-the-Harvest Program. Of the 109 homes to receive geese, 90 homes participated in the interview process for an 83% participation rate; 19 homes were unable to participate in the interviews for several reasons (e.g., the participant was in the hospital, the participant was out of town). Forty-two females and 50 males were interviewed from 90 homes; in 88 homes, only one person was interviewed; while in two homes, husbands and wives were interviewed. Results were shared with First Nation leadership (e.g., Chief, Councilors, and Health Director).

### 3.2. Sharing Networks 

#### 3.2.1. Sharing-the-Harvest: A Regional Initiative in the Cape Henrietta Maria Area, Western Hudson Bay Region, Ontario

**Figure 3:**
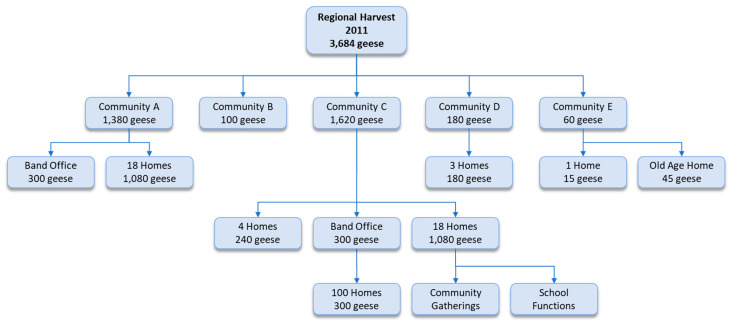
The lesser snow goose (*Chen caerulescens*) sharing network for the regional Sharing-the-Harvest program in 2011. (Note: The 164 geese eaten at camp and 180 geese distributed to another community south of James Bay are not represented, in the sharing-network figure.).

In 2011, a total of 3684 snow geese were harvested and shared with all five western James Bay communities (Figure 3). End-point-level distribution is described for Community C and E. However, end-point distribution was not recorded for Community A, B, and D. In Community C, 1620 snow geese were shared with 122 homes (Figure 3). Taking into account that there were 161 homes in Community C [62], the sharing coverage rate was 76% (i.e., 122 of the 161 homes at a minimum). 

It should be noted that 164 snow geese were eaten at the harvest camp, and 180 snow geese were distributed to a sixth community south of the James Bay region. The aforementioned geese are not included in Figure 3.

#### 3.2.2. Sharing-the-Harvest: A Local Initiative in the Western James Bay Region, Ontario

**Figure 4:**
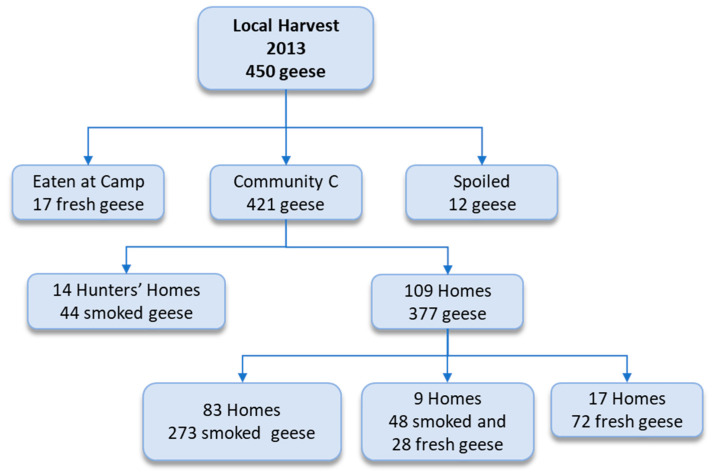
The giant Canada goose (*Branta canadensis maxima*) sharing network for the local Sharing-the-Harvest program in 2013.

In 2013, a total of 450 giant Canada geese were harvested, and shared in Community C (Figure 4). End-point-level distribution in Community C is presented in Figure 4. The 450 giant Canada geese were shared with 123 homes (Figure 4), for a 76% coverage rate. The 12 spoiled geese were used as bait by trappers (Figure 4).

### 3.3. Themes: Regional Sharing-the-Harvest Program

Due to relatively small sample sizes for some of the parameters (e.g., sex, community), selected quotes do not include identifiers to preserve anonymity. It should be emphasized that the themes generated were not all mutually exclusive. 

#### 3.3.1. Transfer of Indigenous Knowledge

Older adult participants expressed the importance of teenagers (18-19 years of age for this program) and youth (up to 24 years of age) being on the land to gain knowledge and experience through vertical transmission (i.e., intergenerational transference) (Table 1). The importance of on-the-land programs was also mentioned, especially in the context of providing opportunities for youth who have encountered several barriers (Table 1). Vertical transmission of knowledge was also seen as being important between Elders and adults. The adults even consulted with the Elders about why the goose harvest was so poor in 2012 (Table 1).

#### 3.3.2. Sharing Aspects

Participants emphasized that the act of sharing is an important part of Cree culture. They also described sharing as being both familial, and beyond familial associations (Table 1).

#### 3.3.3. Social Aspects

Interacting with other participants was identified as an important aspect of their time on the land. Some individuals were very generic in their responses: “I like doing everything with people.” Meanwhile, some were very specific that they enjoyed making new friends (Table 1), and partaking in the camaraderie: “We had a lot of fun […] I was in a group that really liked to laugh.” Others enjoyed being with old friends, and one longed for their company again (Table 1), 

#### 3.3.4. Being on-the-Land

Being on-the-land was identified as being one of the most important aspects of the Sharing-the-Harvest intervention for a variety of reasons. One reason mentioned by a participant related to health: “I like being there [on-the-land] and felt healthy. […] I like to go into the bush.” Other responses specifically referred to the pristine nature of the Hudson Bay Lowlands and the topography (Table 1). 

Of particular interest were responses from participants after the 2012 harvest, because this harvest was relatively unsuccessful, as climate change impacted snow goose migration routes. Interviewees mentioned the low number of geese harvested—and the main reason for the program was the harvesting of snow geese. However, participants still mentioned positive aspects of being on-the-land. For example: “Weather, not many birds but being with people and just being there was good.”; and “I really like hunting [even if unsuccessful], it’s fun there.”

One participant noted that it “was good for the hunters that wanted to go there [but could not because of barriers], go and hunt that never went there before.” Indeed, participants who had never visited the northern reaches of their ancestral territory expressed their positive assessment of their first time experience (Table 1). Other people expressed their happiness of being back home (Table 1), some, perhaps for their last time (Table 1).

#### 3.3.5. Breaking Down Barriers

Participants expressed happiness with the assistance in the form of transportation, hunting equipment, and consumables that were received to break down barriers to people being on the land (Table 2). 

### 3.4. Themes: Local Sharing-the-Harvest Program

#### 3.4.1. Transfer of Indigenous Knowledge

The intergenerational transfer of Indigenous knowledge to youth and beyond the harvest program was mentioned by participants. For example,

“And maybe they [youth] can learn from that [what we have done in the program], so they can experience the bush to learn from it. What we’re doing. What we’re trying to do. Maybe they’ll look forward to it too. Pass it on to those younger kids, to the next generation.”

Furthermore, the issue of Indigenous knowledge transfer was of such importance that one participant suggested that harvest programs should be included in the school curriculum.

“For the young people, you should have it [a traditional harvesting program on-the-land] in the schools.”

The knowledge sharing was not only vertical but also horizontal (i.e., intragenerational).

“I think about the guys; the stories are good—good stories—interesting stories. And we talked mainly about the past. They were talking about the past. […] They were all telling stories and stuff. They were laughing, talking about hunting way back then—you know hunting these days, everything was changing.”

#### 3.4.2. Sharing Aspects

Participants noted the importance of sharing food from a food security perspective.

“It [Sharing-the-Harvest program] benefits my household and the community member here. It does that because of the high cost of living. You cannot purchase anything.”

“It’s good to share the food which you kill, to the community. And we have fun, like helping out each other. We help out each other at the community harvest and try to get as many birdies as we can to share. That’s what’s good about this harvest.”

#### 3.4.3. Social Aspects

The social aspects described included the experience of working together, and with people that participants would not normally associate with in the community.

“And good experience too working together with other people. Building up skills.”

“Going out to be there—some of the guys in town wouldn’t talk to them—talk to each other. Some guys wouldn’t talk to them. But when you’re out there, everybody gets to know everybody. And I say [name removed], this guy is real [name removed], and they say this guy here, he’s somebody that no one talks to. This guy was smart. […] He’s a good guy, a very good guy. Everybody found him out there, and we went there, and went, ‘Oh, he’s a good guy, a really good guy.’ So, it was fun.”

#### 3.4.4. Being on-the-Land

The wellness aspects of being on-the-land were articulated by participants. Further, the barriers faced by single-parent families and women in accessing the land were emphasized.

“Going out there, the fresh air, looking at birds, the birds, observing them, seeing them, and eating them. […] I haven’t been out there for a while, I don’t have a canoe, stuff like that. It was good to go out, and would be better too for general people, because I know that there are a lot of families out there that don’t have income to buy a canoe, engines, and shovels. But if you go out there and experience [the land] it is really good you know. […] That’s the main thing there, because I remember growing up in Moosonee, way back, and same thing. I wanted to go out hunting, but I didn’t have a father living at home, so you know it was hard. […] Oh yeah. It would be good for a woman too. It would be good for a woman to go out there. I know there is always women that would like to go out to the grounds, the hunting grounds, the thing is again, barriers.”

Even when there were hardships on the harvesting excursions, the positivity of the experience comes through in the end assessment.

“So, we slept in tents two nights, and all the other nights we slept [in the boat]. It was tough. It was very tough. Brutally, pretty tough, yeah. We got some [birds] but it wasn’t much. [But] Fun to be out there—a lot of fun, just fun to be out there with the guys there—a bunch of guys going out there talking about anything. […] I told [name removed] that going out there is going to be really good for you, you’ll have a good rest, no one yelling at you up there. No one yelling at you is going to be restful, just peaceful. It’s going to nice out there. Going to sleep good is what I was telling him there. But, when we go out there, first night—first day was the roughest. We couldn’t find the river, the creek, the wood. So, we got stuck on the boat all night in the mud [tidal flats]. […] I’m guessing it must’ve been zero. Temperature must have been zero. We slept [on the boat], we just covered in tarps. […] Next morning we got up. It started to rain, all day. […] [name removed] stayed in his huddle all day. I never saw him get out once. […] There must’ve been half-a-mile that made the waves rough and it was pitch black. […] We had terrible weather. Just to top it off, we flooded a bit [after we had set up camp]. […] We had a good time after [meeting up with everyone and setting up camp]. ”

#### 3.4.5. Breaking Down Barriers

One of the important objectives of the Sharing-the-Harvest intervention was to break down barriers that were keeping people from harvesting on the land. The importance of assistance in the form of transportation, hunting equipment and consumables was mentioned by the participants (Table 2). In addition, in the future, the harvesting program can be more inclusive of women since many have acquired their Possession and Acquisition License (PALs) through our initiative (Table 2).

#### 3.4.6. Sustainability of the Sharing-the-Harvest Program

Participants identified avenues of funding for the Sharing-the-Harvest program that could potentially contribute to the sustainability of the harvesting initiative (Table 2). The Band (First Nation locally-elected government) was identified as a potential source of direct support for consumables or indirect support through an Impact Benefit Agreement (Table 2). Impact Benefit Agreements (IBAs) are negotiated agreements, typically between Indigenous groups and resource developers, used to secure consent for development projects on Indigenous homelands [63].

### 3.5. Themes: Households Participating in the Local Sharing-the-Harvest Program

#### Increasing Food Security

Participating households overwhelmingly mentioned the increasing food security aspect of the harvest program. What is important to note is that this positive aspect was not related to an individual or household level, but to a “people” or “community”, or “us”, or “all” level (Table 2). Nonetheless, people in need were identified as important end-point beneficiaries (Table 2).

## 4. Discussion

### 4.1. Sharing Networks 

For the James Bay Cree, being Cree is governed by codes of conduct [20]. An important part of the Cree codes of conduct is the sharing of a persons’ harvest [20]. Indeed, the importance of sharing was highlighted in the interviews. Sharing included familial connections, but sharing went beyond the familial, being community-level in nature. The sense of community was reinforced by the harvest sharing, and this is seen by the results of the interviews where participating households mentioned the increasing food security was at the “people” or “community”, or “us”, or “all” level, and especially important for people of low income (Table 2).

Although the sharing of the goose harvest was a provision of the regional and local-level interventions [32,55], the actual sharing between communities was dictated by the harvesters themselves; while, local-level sharing was decided upon by the harvesters, helpers, Elders, and the Band Offices. The extensiveness of the sharing networks are evident in Figure 3 and Figure 4. Noteworthy, in Community C for which we have relatively detailed data, at a minimum 76% of the homes received geese from the harvest for both the regional and local interventions, and the sharing network was in reality even more extensive with geese being shared with the school and at community gatherings (Figure 3).

Referring to the interviews, the harvesters felt good about sharing their harvest with Elders, schoolchildren, and people in need. Harvesters also describe their experience as having “fun, [and we] like helping each other out.” This feeling of community was a thread that appeared in the interviews of both the harvesters and the people who received the geese. Clearly, contributing to community cohesion is a wellness benefit beyond food security improvement stemming from this harvest sharing intervention.

### 4.2. Indigenous Wellbeing Benefits 

It is known worldwide that Indigenous groups have significantly lower standards of health compared to their non-Indigenous counterparts, and although many closing-the-gap health programs have been initiated, this gap remains [64,65]. Recently, there has been a movement away from this deficit model with more attention being paid by governments and researchers to wellbeing and the importance of land (which includes water and air) to Indigenous people—and more specifically the importance of time on-the-land as related to health and wellbeing (wellness) [66,67].

Worldwide, wellbeing has been suggested to exist in two dimensions: the objective (relatively easy to measure) and the subjective (more difficult to measure) [68]. The objective dimension includes singular surrogate measures (e.g., Gross Domestic Product) [69,70] and composite measures (e.g., the Human Development Index) [71]. Health is seen as a component of wellbeing in the composite measures [68,69]. In contrast, the subjective dimension of wellbeing has been reported to be composed of an individual’s experience of their life [72] and has two main components, supportive relationships and a feeling of belonging, which are important for a happy life [69]. In Canada, the Community Well-Being Index was developed to measure the wellbeing of individual Canadian communities using four parameters (i.e., education, labor force activity, income and housing) to derive a community wellbeing score [71]. These scores were used to compare wellbeing across Indigenous (First Nation, Inuit, and Metis) and non-Indigenous communities over time [71]. It is well recognized that this index was flawed, since there was no consultation with Indigenous people and culture, language, and other variables were not included [73]. In 2012, the Canadian Index of Wellbeing was initiated in an attempt to measure subjective wellbeing in Canadians [74]. This composite measure was based on core values identified in consultation with non-Indigenous Canadians [74]. Similarly, in Australia, nationwide consultation yielded a composite measure of wellbeing, the Measures of Australia’s Progress [75], without Indigenous peoples’ input. Other levels of Indigenous wellbeing occur at the familial and individual levels [72]. In Australia, it is recognized that for Indigenous people “country” is central to wellbeing [76]. Likewise, Alaska Natives use the phrase “keeping busy” to describe wellbeing (and health), in the context of being on the land eating traditional (“country’) food and respecting Elders and nature [77]. Other Indigenous descriptions of wellbeing (and health) incorporate the medicine wheel [78]. In Canada, Adelson [79] has described Cree wellbeing (and health) as “being alive well”; that is, being on the land and connected to everything that makes a person Cree (e.g., partaking in traditional activities). 

#### 4.2.1. Transmission of Indigenous Knowledge and the Strengthening of Social Linkages

As with other Indigenous communities worldwide, sharing goes beyond food; sharing of knowledge is also of importance to Indigenous wellbeing [80]. In the western James Bay region, significant intergenerational loss of Indigenous knowledge has been reported [81] and concern over this loss expressed by Cree Elders and people with extensive experience of being on the land and following Cree codes of conduct [20]. Although youth benefited by gaining experience of being on-the-land in the present harvest program, older harvesters identified the need to provide more on-the-land opportunities, so that additional teenagers and youth can gain on-the-land knowledge and skills through experiential learning. Experiential learning is foundational for Indigenous learning [82], and well received by Cree youth [32,83]. Vertical transmission of knowledge was not only between Elders and youth, but also between Elders and adults (e.g., the Elders offered an explanation as to why the snow goose harvest was relatively unsuccessful in 2012; Table 1). There was also knowledge transmission within a generation as evident in the 2013 harvester interviews; harvesters related past exploits and stories with their peers, putting their experiences in the context of present-day environmental change. The camaraderie between the harvesters was discernable.

Clearly, participants enjoyed being with other people and working as a group; these social aspects were identified as important benefits of their time on the land. Moreover, participants reported that making new friends and/or renewing old friendships was important (Table 1). Interestingly, being on-the-land with a common objective brought people together who had never associated with each other before the harvest program; that is, being on-the-land together broke down social barriers erected in the First Nation community. Thus, the benefit of being on-the-land was that this activity fostered supportive relationships and a feeling of belonging, which are important components of subjective wellbeing.

#### 4.2.2. Being on-the-Land

Recently there has been a trend to include Indigenous cultural activities in health and wellness initiatives, because the potential benefits of Indigenous cultural activities go beyond physical health (e.g., contributing to wellbeing) [84,85,86,87,88]. Positive outcomes have been associated with a variety of Indigenous cultural activities worldwide, such as, hula dancing [89], Indigenous games [90], gardening [91] and going back to country [92]. For Indigenous Australians, “country” is a living entity and multidimensional [93], and caring for country means participating in activities that promote environmental, spiritual, human health, and wellbeing benefits [94]. However, studies with Inuit using self-reported health and wellbeing data have revealed little correlation with time on the land and wellbeing measures, or only minor differences in health perception between those on the land and those not on the land [95]. By contrast, a significant positive relationship between cultural continuity (which includes land-based activities) and mental health (e.g., suicide rates) has been shown [96,97,98]. The difficulty in studying health and wellbeing in the context of land-based activities has been noted in a recent review [99].

In Canada, the importance of land/bush (this term includes water) to Indigenous health and wellbeing has been documented [100,101]. Barriers to being on the land can negatively impact Indigenous health and wellbeing through a multitude of ways, such as, but not limited to: not being able to practice and learn Indigenous knowledge; no opportunity to engage in social relationships that foster healthy behaviours; and decreased access to traditional foods [102,103,104]. When barriers to being on-the-land are removed − such as in the present harvest program − there are opportunities to transmit Indigenous knowledge, partake in positive social interactions, and participate in the harvest and consumption of traditional foods. In the present harvest program, being on-the-land was identified as being very important to health and wellbeing. Even when the snow geese were not flying in the spring of 2012, harvesters still relayed a positive experience during the interviews, such as: “it’s beautiful […] it was fun being there”; “Always wanted to go there. Go again if I could”; and “Like going home. Not sure do it again, age I guess.” (Table 1). Even when one harvester states that—“It was tough. It was very tough. Brutally, pretty tough, yeah”—he still ends with “We had a good time after [meeting up with everyone and setting up camp].” Thus, being on-the-land can be challenging both physically and mentally, but the benefits outweigh any of the challenges. 

### 4.3. Sustainability of the Sharing-the-Harvest Program

The Sharing-the-Harvest initiative broke down barriers to being on-the-land and partaking in the Cree lifestyle. However, to be part of the intervention as a harvester, a person had to possess a valid Possession and Acquisition License (PAL). This requirement was problematic, because the PAL course was rarely conducted in remote First Nations; in addition, there were economic (e.g., the cost of the PAL course; licensing fee), and literacy (e.g., the written test was in English or French) constraints [105]. In Canada, although First Nation people do not need a hunting or fishing license to partake in subsistence pursuits, First Nation people do require a Government of Canada PAL to allow for the legal purchase and possession of firearms and ammunition [105]. To address this barrier, the research team and the Royal Canadian Mounted Police provided assistance with a licensing drive in two of the James Bay First Nations that allowed more than 100 people to obtain their PAL [105].

The PALs and equipment from the harvest program have allowed the harvest initiative to continue beyond the duration of the original program. For example, in 2014 after the regional harvest program ended, some harvesters paid their own way back to Cape Henrietta Maria. The harvesters used the snowmobiles and tents supplied by the harvest program—because this equipment was left for the communities and people to use after the program—and shared their harvest of an estimated 1000 snow geese and 500 incidentals (i.e., other geese species and ducks) with others [32]. Similarly, since 2015, local hunters from Community C have been harvesting ~500 giant Canada geese annually to share with their community [32]. Nevertheless, even with the equipment and PALs, the continuation of the harvest program requires some monetary input. Thus, participants have suggested that the Band provide assistance (e.g., gas) and/or using the IBA to sustain the harvest program in the future (Table 2). In the western James Bay First Nations’ IBAs, there was a provision for harvesting activities [63]. Noteworthy, the participants are not so much offering suggestions for sustaining the harvest program per se, but for sustaining Cree culture.

### 4.4. Limitations

Although the sample sizes were relatively robust in the present study, participants were overwhelmingly male, with only two harvesters being female. This oversampling of males is related to the fact that Cree hunters have been and are usually male, with females, rarely, historically speaking, possessing PALs [105]. However, through the Sharing-the-Harvest program future initiatives can be more inclusive of women, because as noted by one participant: “All the wives have their, got their [PAL] licenses.” (Table 2). Another limitation of the study is related to the transcription of interviews when interviewees spoke in Cree, because some Cree words and concepts are not directly translatable to the English language. In addition, Elders spoke “high” Cree, not just conversational Cree, which makes translation to English even more difficult. However, our translators were fluent in high Cree. Nonetheless, we have indicated which quotes were an “English translation from Cree” in the article.

### 4.5. Transferability

Worldwide, the model of wildlife conservation has been evolving to address wildlife overabundance [106], and invasive species [107]. Overabundant and invasive species around the world need to be managed before they further damage the environment [32]. Overabundant species exist at population levels that negatively impact one or more of the following: their own habitat; the environment of other species; the economic interests of individuals and groups of people; and human health [22,32,108]. There are other overabundant species in the world other than geese that could be part of an Indigenous harvesting program, where food security issues could be addressed along with environmental sustainability, and culturally-important benefits could also be realized.

It should be noted that the Sharing-the-Harvest program described in the present paper was specific to the James Bay Cree of subarctic Ontario, Canada; nonetheless, our protocol could be suitably modified for other overabundant game species. For example, around the world (e.g., Australia, Europe, Japan, New Zealand, North America, South America), native and/or invasive deer species are impacting environmental integrity [32,109,110]. In North America, white-tailed deer (*Odocoileus virginianus*) are overabundant [106], due to anthropogenic factors (e.g., extirpation of large predators of deer, direct feeding) [106,111]. There are many impacts of overabundant white-tailed deer: habitat destruction [111]; deer-vehicle collisions leading to vehicular damage [112] and human deaths [113]; increased risk of Lyme disease in humans [113]; and damage to agricultural crops [113]. Hunting has been identified as the primary means of addressing the overabundance of white-tailed deer [106]. Thus, venison donation programs for marginalized populations have arisen to help combat food insecurity, and contribute to wildlife conservation in North America [106,114,115]. However, similar to geese harvested with lead shotshell, deer harvested with lead bullets contaminate the harvested game meat [106,116]. Approximately 6% of donated venison samples [117] have been reported to be unfit for human consumption. Additionally, an association between elevated blood-lead levels in humans and wild game consumption has been noted in North America and Europe [118,119]. Thus, there is an opportunity for deer harvesting programs to include Indigenous people worldwide, as long as lead-free ammunition is used, and lead-free ammunition is available in a variety of calibers [120,121]; or bow hunting could be employed [122].

Additionally, in the USA, it has been suggested that the overabundant and invasive fish species with the designation “Asian Carp” be harvested to address food security issues for low-income Americans [123]. The harvesting of Asian Carp would supply a major source of protein and omega-3 fatty acids for the marginalized—and the fish tissue is said to be light, flaky (albeit with bones), and mild tasting [123] − but there have been concerns about the bioaccumulation of toxins in Asian Carp [107]. However, it has been reported that contaminant concentrations (e.g., mercury) in Asian Carp were generally low, but individual variation between fish in contaminant burden was noted [123], probably related to variation in fish size and location of fish harvest. In summary, opportunities exist for harvest sharing programs of overabundant and/or invasive wild game and fish to address food security issues among marginalized groups worldwide, including Indigenous peoples, and the benefits for some groups would be more than just addressing food security and environmental sustainability issues.

## 5. Conclusions

Our harvest program allowed the Cree to participate in traditional on-the-land activities—including the sharing of a relatively uncontaminated, nutritious source of game meat − decreasing food insecurity in the subarctic communities [32]. By harvesting overabundant geese known to detrimentally impact the environment, the harvesters helped in part to address a food insecurity issue, but also contributed in part to the effort to protect and provide time for the recovery of impacted ecosystems [32]. In addition, the harvest program also had benefits beyond addressing food insecurity and environmental sustainability issues (i.e., protecting the environment for future generations). For example, the harvest program allowed for the practicing of the important Cree custom of sharing, the transmission of Indigenous knowledge, the strengthening of social networks, and other wellbeing benefits associated with being on-the-land (e.g., happiness). Thus, Indigenous people would gain benefits beyond those solely related to food insecurity and environmental sustainability, if participating in an on-the-land harvest program.

Furthermore, any food insecurity intervention which includes a harvesting and/or gathering (e.g., berries, wood) component must be based locally—not regionally—especially in rural and remote regions of the world, for several reasons [32]. First, when programs are based locally, the timing of on-the-land activities can be quickly changed to adapt to challenges using Indigenous knowledge [32]. The second major factor relates to the cost of travel in rural and remote regions of the world, which can be expensive, making budgeting very difficult [32]. Taking into account the above-described caveats—and that overpopulated and/or invasive species occur elsewhere—our Sharing-the-Harvest protocol can potentially be adapted and employed by other Indigenous (or marginalized) groups worldwide, to improve health and wellness [32]. Lastly, the importance of time spent on the land is important not only to Indigenous peoples, but also to non-Indigenous populations with respect to health and wellbeing (e.g., “Green Gym,” [124,125,126,127] and “Blue Gym” [128,129,130]).

## Figures and Tables

**Table 1:** Themes and subthemes of the Regional Sharing-the Harvest Program directly related to benefits other than those related to food security and environmental sustainability.

Themes	Subthemes	Representative Quotes
Transfer of Indigenous Knowledge	Vertical Transmission (Elders and Youth)	“*Missing young teenagers [< 18 years of age]. Two [≥ 18 years of age] were working, nice to have more, so learn.*” “*They [youth] need to know the [traditional] way. Experience hunting, the land, different types of animals, like caribou. […] To get more experience.*” “*Bring more youth there. […] Concentrate on the youth that don’t have an opportunity under normal circumstances, maybe they don’t have parents, maybe they never get to go out on the lands. Maybe they need to go out there, the ones that don’t get to go out, maybe, concentrate on those types of youth and give them that opportunity. We have youth like that in all our communities, ones that are never [on the land], that don’t have uncles, or brother, or parents to take them out. And they’ve never experienced that. So we should concentrate on those youth, maybe that are at risk. […] not dangerous ones, but ones that need help like that, and maybe bring them out and it can be a learning experience for them too and to leave it open for other people as well.*”
	Vertical Transmission (Elders and Adults)	“*I learned a lot from them, when I was there. I learned a lot from them, like the Elders that were there. They have experience, they’ve been there so many years; I learned a lot from them.*” “*Elder said that because no snow [2012], early spring, wavies [snow geese] flying along the shoreline of the bay [Hudson Bay]. Also, said that is why they are [flying] high because no snow.*”
Sharing Aspects	Familial and Extrafamilial	“*[Sharing] that’s the main thing we’re supposed to do, we’re supposed to share our hunt. […] I mean myself, our brother in-laws, we share our [harvest…] to the Elders you know. And of course the Elders want to pay us, and I tell them, ‘No, we don’t want any money, we just want to give it to you.’*” “*I already give my geese, 50% away when I get here previous years [when not part of the Sharing-the-Harvest program]. That’s half of my geese I was going to give when I land here anyways. So I didn’t need to make a special effort to [share my geese].*” “*When I come home with sixty birds [their share from the Sharing-the-Harvest intervention] that’s too many for me. So I just give them to family and friends and that type of stuff. And to schools and just to people in need.*”
Social Aspects	Making New Friends	“*We met a lot of new friends, when we were there.*” “*I like when I went there, yeah!. I like meeting new people.*”
	Seeing Old Friends	“*I like seeing my friends. […] I like seeing people there I haven’t seen for a long time.*” “*I have a good time staying over there. So when I come home I’m lonely and thinking about all the time after that.*”
Being on the Land	Enjoying the View	“*Like it there. Land is so nice, clean. I like it flat. Went onto the bay [Hudson Bay], two-and-a-half hours onto the bay, saw caribou.*” “*Go check out the scenery. […] It’s [Hudson Bay] lowlands. […] A little different I think than over here [James Bay].*” “*I like being there, it’s beautiful, seeing animals, caribou and wavies [i.e., snow geese]. Not enough wavies, but it was fun being there.*”
	New Experiences	“*Good fun. Liked it. Everything was good living out there, first time. Like spring camp, same thing.*” “*Windy, cold, and no geese flying. [But] Nice to get away. I liked the land, first time I had been there. […] Eat ptarmigan, caribou, Canadas and wavies [lesser snow geese]. Always wanted to go there. Go again if I could.*”
	Familiarity of Being Back Home	“*I always enjoy it, to go over there, that’s it [English translation from Cree].*” “*I really like going there [English translation from Cree].*” “*First time been back [since the 1960s] [English translation from Cree].*” “*Happy even no geese. Walking around […] Like going home. Not sure do it again, age I guess [English translation from Cree].*”

**Table 2:** Themes not directly related to benefits accrued while being on the-land.

Theme	Harvest Program	Representative Quote
Breaking Down Barriers	Regional Initiative	“*The benefits like the food and the ammunition and the firearms, and the accommodations, and the tents […] I am very, very, very happy about the privilege of having gone up there, and some costs being taken care of, that doesn’t happen a lot.*” “*Yes, I like it and it’s good that there are ski-doos [snowmobiles] there [for use in the future, after the regional initiative ends].*”
	Local Initiative	“*The economy is so low here. A lot of people eat that kind of stuff [traditional foods], But they can’t afford to buy gas to go get the food. And they think it’s too expensive.*” “*The harvesting program, what helped a lot was the equipment, like a shotgun. I had no shotgun before. Every time I go out, I have to borrow a shotgun. What helped also was getting my FAC [the previous designation for the Possession and Acquisition License]. It expired a long time ago. Couldn’t go hunting. So, it helped bring the food in for the family and enjoy having dinner with them.*” “*I checked with the wives. All the wives have their, got their [Possession and Acquisition] licenses.*”
Sustainability of the Harvest Program	Local Initiative	“*[The Band has in the past helped] with purchasing gas [to go into the bush]. That would prolong our Native [Indigenous] way of life.*” “*Funding would be probably through IBA [Impact Benefit Agreement] because there’s probably money for that kind of thing—harvesting. And that would be one way to get money [to sustain the harvesting program] would be IBAs. […] Just to keep it that way, for other generations that come behind us, to learn how to use a gun—for safety wise—and learn to respect the animals. […] Cause to have it running would benefit the community; there would always be food coming in, and if we had some kind of organization going for hunting [Canada] goose, or wavy [snow geese] hunting every year—you keep it going—even the generation behind us can start doing it, if they want to do it that way. The IBA money would be a lot of money for this kind of thing, I think.*”
Increasing Food Security	Local Household food sharing network	“*This program Sharing-the-Harvest is good for people that are short of food. It helps.*” “Good for community.” “*A good program for us.*” “*A good program for all.*” “*The extra meat is a plus. The price of food is high. The program Sharing-the-Harvest helps low income families.*”
